# Recent advancement in finite element analysis of spinal interbody cages: A review

**DOI:** 10.3389/fbioe.2023.1041973

**Published:** 2023-03-23

**Authors:** Ruofan Wang, Zenghui Wu

**Affiliations:** ^1^ Guangzhou Key Laboratory of Spine Disease Prevention and Treatment, The Third Affiliated Hospital of Guangzhou Medical University, Guangzhou, China; ^2^ Department of Orthopaedic Surgery, The Third Affiliated Hospital of Guangzhou Medical University, Guangzhou, China

**Keywords:** finite element analysis, interbody cage, spinal fusion, biomechanics, modeling

## Abstract

Finite element analysis (FEA) is a widely used tool in a variety of industries and research endeavors. With its application to spine biomechanics, FEA has contributed to a better understanding of the spine, its components, and its behavior in physiological and pathological conditions, as well as assisting in the design and application of spinal instrumentation, particularly spinal interbody cages (ICs). IC is a highly effective instrumentation for achieving spinal fusion that has been used to treat a variety of spinal disorders, including degenerative disc disease, trauma, tumor reconstruction, and scoliosis. The application of FEA lets new designs be thoroughly “tested” before a cage is even manufactured, allowing bio-mechanical responses and spinal fusion processes that cannot easily be experimented upon *in vivo* to be examined and “diagnosis” to be performed, which is an important addition to clinical and *in vitro* experimental studies. This paper reviews the recent progress of FEA in spinal ICs over the last six years. It demonstrates how modeling can aid in evaluating the biomechanical response of cage materials, cage design, and fixation devices, understanding bone formation mechanisms, comparing the benefits of various fusion techniques, and investigating the impact of pathological structures. It also summarizes the various limitations brought about by modeling simplification and looks forward to the significant advancement of spine FEA research as computing efficiency and software capabilities increase. In conclusion, in such a fast-paced field, the FEA is critical for spinal IC studies. It helps in quantitatively and visually demonstrating the cage characteristics after implanting, lowering surgeons’ learning costs for new cage products, and probably assisting them in determining the best IC for patients.

## 1 Introduction

Finite element analysis (FEA) is a widely used tool in many industries and research activities that decomposes complex individuals into a finite number of units and interconnected nodes and then computationally controls these units and nodes to study the properties of the individuals. The method was first developed in the aircraft industry in the 1950s and has since been adopted in a variety of other fields (Fagan et al., 2002). The first application of FEA in biomechanics was probably published in 1972 by Brekelmans et al. (1972). Since then, Belytschko et al. (1974) developed the first finite element models of the spine by applying FEA to spine biomechanics. FEA has contributed to the understanding of the spine, its components, and its behavior in healthy, diseased, or damaged conditions over the last few decades, supplementing *in vitro* experiments (Fagan et al., 2002). Furthermore, they aid in the prediction of surgery outcomes for the most stressed parts of spinal units, which are prone to damage, at low cost and with no risk to patients. FEA has undeniably become a common research method in the field of *in silico* medicine (Viceconti et al., 2008).

FEA has gradually been applied to various spine-related research with the development of finite element models of the spine, one of which is the spinal ICs. ICs are a highly effective instrumentation for achieving fusion that has been used to treat a wide range of spinal disorders such as degenerative disc disease, trauma, tumor reconstruction, and scoliosis (Jain et al., 2016). They help to restore intervertebral height, enable bone graft containment for arthrodesis, and restore anterior column biomechanical stability. However, the spine is a very complex structure, and the development of an interbody cage is fraught with uncertainties. At this point, the use of finite element models to somewhat simplify and idealize the problem is frequently a strength, allowing new designs to be thoroughly “tested” before a cage is even manufactured, bio-mechanical responses and fusion processes that cannot easily be experimented upon *in vivo* to be examined, and “diagnosis” to be performed (Fagan et al., 2002). In recent years, many reviews have summarized and studied the materials and design of ICs, as well as the corresponding fusion techniques (Phan and Mobbs, 2016; Enders et al., 2020; Gou et al., 2021; Macki et al., 2021; Tan et al., 2021). However, few reviews concentrate on the FEA of ICs. Fagan et al. (2002) reviewed FEA in spine research and considered its positive support in spinal instrumentation design and application. Jain et al. (2016) reported advances in spinal ICs from 2013 to 2015, highlighting trends in cage design optimization, materials, bone graft alternatives, and coatings that may improve fusion. They found that FEA studies can be used to identify theoretical qualities to try to incorporate when designing the next-generation of cages. With more progress and refinement of FEA research on ICs, a review is needed for a dedicated summary.

Therefore, the purpose of this paper is to categorize, summarize, and review the latest FEA studies of ICs conducted over the last six years (2016–2021) to provide an update on recent research findings involving cage materials, cage design, fixation devices, bone formation, fusion techniques, and pathological structure influence. Furthermore, the modeling methods and limitations discussed were summarized and the relative prospects were proposed.

## 2 Modeling methods and public database resources

**FIGURE 1:**
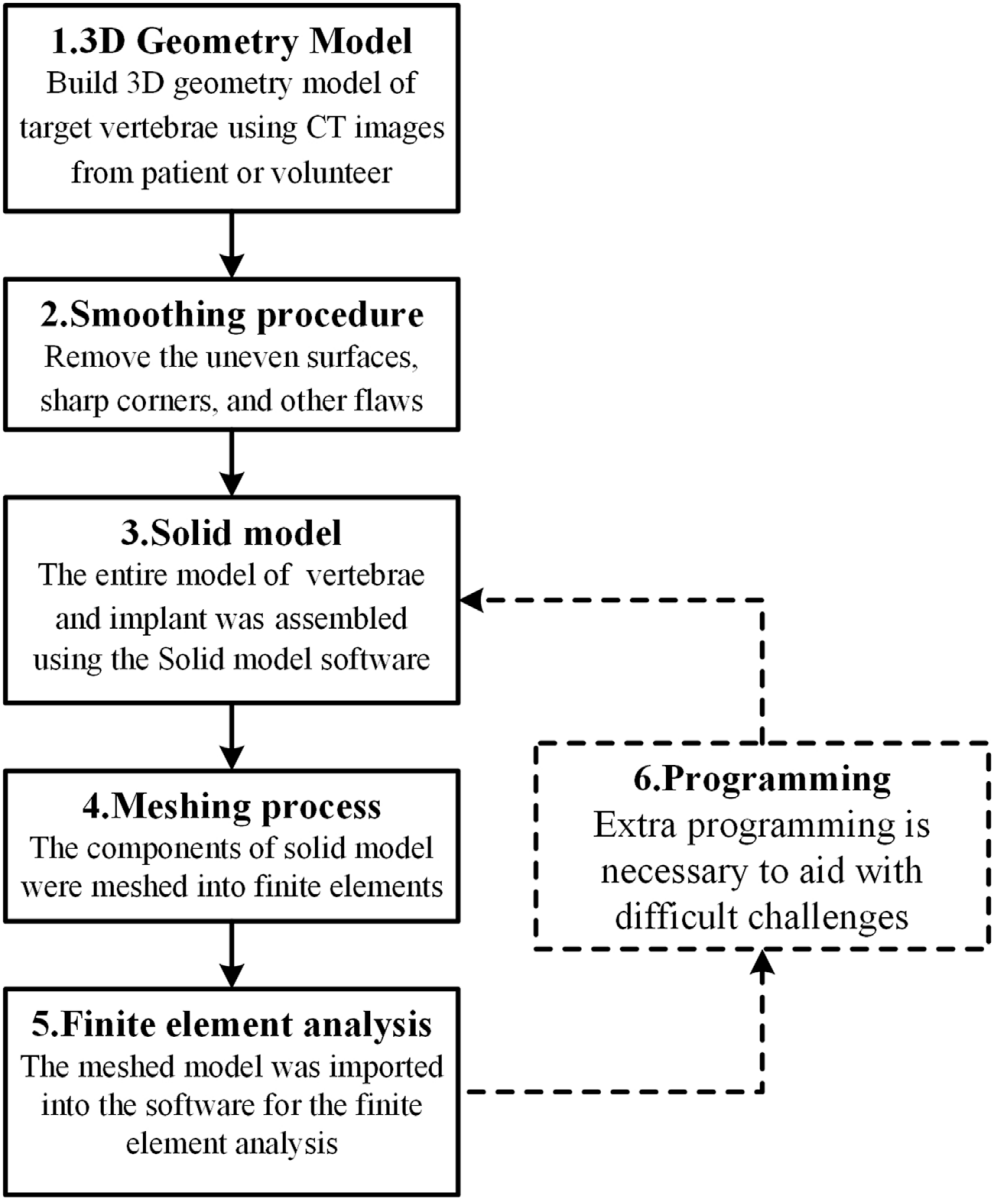
A summary of the modeling process flow.

The IC finite element model is normally made up of three parts: the cage, the fixation device, and the spine. With the development of FEA technology, the modeling methodologies and simulation processes of the IC FE model have steadily improved. The following is an overview of the modeling process flow as represented in Figure 1: Image processing methods were commonly used to generate the 3D geometry model of the target vertebrae using computed tomography (CT) images of a patient or a healthy volunteer. The uneven surfaces, sharp edges, and other faults were subsequently addressed using a smoothing procedure. The cage and internal fixation were then constructed, and the entire model of the vertebrae and implant was generated using solid model software. The meshing tool was used to transform the solid model components into finite elements, which were then imported into finite element analysis software for analysis. In addition, extra programming tools are necessary to aid with challenging tasks such as bone formation and cage topology optimization.

**FIGURE 2:**
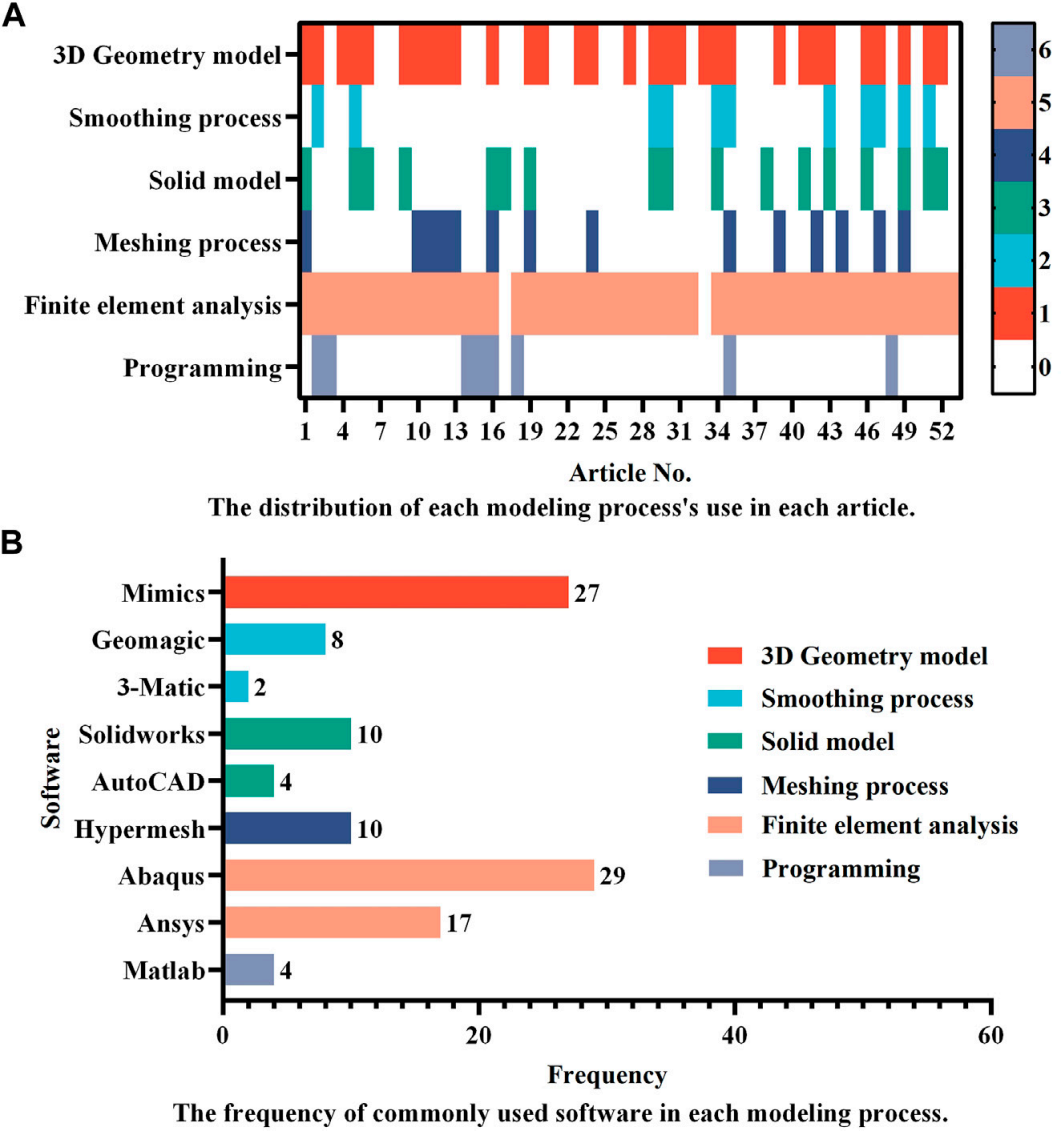
A summary of **(A)** the distribution of each modeling process’s use in different articles and **(B)** the frequency of commonly used software in each modeling process. Studies that did not provide detailed descriptions of the modeling process and software were excluded, leaving 53 articles for statistical analysis. In **(A)**, the numbers in the colormap represent the process order depicted in Figure 1, with the number “0” indicating that the process is omitted.

Finite element analysis should be based on correct modeling. Consequently, the complete modeling process is the most recommended. However, in practice, it is permissible for certain modeling steps to be removed owing to computational efficiency considerations. According to the use distribution of each modeling process as shown in Figure 2A, the 3D geometry model and finite element analysis seem to be the two most essential procedures in the FEA of ICs. In addition, scientific software selection is equally critical for accurate modeling. Therefore, the frequency of regularly used software in each modeling process is also provided in Figure 2B. According to the statistics, Mimics (Materialise, Inc., Leuven, Belgium) is the most extensively used software for 3D geometric models. The following software: Geomagic (Geomagic, Inc., North Carolina, United States), Solidworks (Solidworks, Inc., Massachusetts, United States), and Hypermesh (Altair Technologies, Inc., California, United States) are the most commonly used software for the smoothing process, solid modeling, and meshing process, accounting for 8, 10, and 10 applications, respectively. Accounting 29 and 17, respectively, show that Abaqus (Simulia, Inc., Rhode Island, United States) and Ansys (Ansys, Inc., Michigan, United States) are the two most used finite element software, proving their relevance in related research. Only a few researchers employ programming software, with Matlab software tools being the most popular. Abaqus and Ansys, on the other hand, have their own programmable language components, Abaqus subroutine UMAT and Ansys APDL, which may be used to program simulations. These popular software programs assist in guaranteeing the relative accuracy of study outcomes and are appropriate for researchers undertaking relevant investigations.

The assignment of material properties is crucial before the final FEA is performed. The cage and fixation device are generally made of a single material, such as titanium or PEEK, whereas the spine is made up of vertebral bodies (cortical and cancellous bone), posterior bodies, intervertebral discs (annulus ground substance and nucleus pulposus), and seven spinal ligaments (i.e., anterior longitudinal, posterior longitudinal, flavum, supraspinous, interspinous, intertransverse, and capsular ligaments). As a consequence, the material properties of the spine component in the IC finite element model vary, and the most frequently used in the cervical and lumbar spines are illustrated in Tables 1, 2, respectively.

**TABLE 1:** Material properties of cervical FE model components that are most frequently used in 9 cervical cage articles.

Material	Elastic modulus (MPa)	Poisson ratio	Section-area(mm^2^)	Number of articles
Cortical bone	12000	0.29	-	6
Cancellous bone	450	0.29	-	5
Posterior body	3500	0.29	-	4
Annulus ground substance	3.4	0.4	-	4
	4.2	0.45	-	3
Nucleus pulposus	1	0.49	-	7
Ligament				
ALL	30	0.3	6.1	3
PLL	20	0.3	5.4	3
SSL	8(<20%), 15(>20%)	0.3	30	1
ISL	1.5	0.3	13.1	2
ITL	10(<18%), 58.7(>18%)	0.3	1.8	1
LF	1.5	0.3	50.1	3
CL	20	0.3	46.6	3

ALL: Anterior longitudinal ligament, PLL: Posterior longitudinal ligament, SSL: Supraspinous ligament, ISL: Interspinous ligament, LF: Ligamentum flavum, ITL: Intertransverse ligament, CL: Capsular ligament.

**TABLE 2:** Material properties of lumbar FE model components that are most frequently used in 42 lumbar cage articles.

Material	Elastic modulus (MPa)	Poisson ratio	Section-area(mm^2^)	Number of articles
Cortical bone	12000	0.3	-	41
Cancellous bone	100	0.2	-	34
Posterior body	3500	0.25	-	28
Annulus ground substance	4.2	0.45	-	17
	Mooney–Rivlin C10 = 0.18, C01 = 0.045	-	-	9
Nucleus pulposus	1	0.49	-	23
	Mooney–Rivlin C10 = 0.12, C01 = 0.03	-	-	8
Ligament				
ALL	7.8(<12%), 20(>12%)	0.3	63.7	10
	20	0.3	63.7	9
PLL	10(<11%), 20(>11%)	0.3	20	10
	20	0.3	20	9
SSL	8(<20%), 15(>20%)	0.3	30	10
	15	0.3	30	8
ISL	10(<14%), 11.6(>14)	0.3	40	10
	11.6	0.3	40	8
ITL	10(<18%), 58.7(>18%)	0.3	1.8	10
	58.7	0.3	3.6	8
LF	15(<6.2%), 19.5(>6.2%)	0.3	40	10
	19.5	0.3	40	11
CL	7.5(<25%), 32.9(>25%)	0.3	30	10
	32.9	0.3	60	8

ALL: anterior longitudinal ligament, PLL: posterior longitudinal ligament, SSL: supraspinous ligament, ISL: interspinous ligament, LF: ligamentum flavum, ITL: intertransverse ligament, CL: capsular ligament; Mooney–Rivlin: Hyper-elastic material model.

Linear and isotropic material properties were adopted for most spinal components in both cervical and lumbar spine models, but non-linear material properties, such as the Mooney-Rivlin hyper-elastic model for annulus ground substance and nucleus pulposus and the bilinear material model for ligament (greater or less than a transition strain resulted in a different elastic modulus), were also used in many studies of the lumbar spine (Table 2). The non-linear material models are superior to linear models in reflecting the true physiological properties of the spine, but at the cost of requiring significant computational resources.

Public database resources, in addition to patients and healthy volunteers, are a valuable source of spine data. It offers the potential to minimize research expenses while enhancing the reproducibility and comparability of outcomes. The Visible Human Project database (National Library of Medicine, National Institutes of Health, United States) is a publicly-available database that comprises anatomically precise 3D data of a human male and female body. An intact lumbar vertebrae FEM model may be generated using the CT scans of the spine from the Vision Man data, and it has been successfully utilized to assess the biomechanical behavior of a novel Apatite-Wollastonite cage (Bozkurt et al., 2018). For other FEA investigations, open source 3D CAD libraries such as GrabCAD and the 3D CAD modeling library (https://www.3dcadbrowser.com) are also appropriate options. The data was effectively used to evaluate the biomechanical effects of a newly designed lumbar cage (Lee et al., 2021) and compare different lumbar interbody fusion techniques (Umale et al., 2021). Another available data alternative is the Mimics 3D lumbar model, which was used to test the biomechanical stability of the Oblique Lumbar Interbody Fusion (OLIF) cage (Fang et al., 2020).

To some extent, the use of public databases and a variety of FEA-related software has helped to lessen the barrier to spine finite element research. Furthermore, as evidenced by the fact that it has progressed from the simple single-segment spine model (Zhang et al., 2016b; Liang et al., 2020) to a complex intact cervical or lumbar spine model (Tsai et al., 2016; Park and Jin, 2019; Fan et al., 2021a), and even a model of the intact spine with rib cage (Jia et al., 2020), the evolution of finite element software has aided the development of the spinal finite element model, which greatly expands the research ranges of FEA in the spine and lays a feasible foundation for solving more practical physiological problems.

## 3 FEA advances in ICs

### 3.1 Cage materials

Titanium alloy (Ti) and polyether ether ketone (PEEK) are the two most common materials used in ICs today. However, Ti is non-degradable and has a much higher elastic modulus than cortical bone, which may cause stress shielding, whereas PEEK is an inert polymer with low biological activity and osteogenic efficiency. As a result, new materials are constantly being created. Different materials have varying mechanical and biological characteristics as shown in Table 3 and FEA can test and verify the effectiveness of innovative material cages while also providing pre-clinical data support.

**TABLE 3:** Mechanical and biological properties of different cage materials.

Material	Elastic modulus (MPa)	Poisson ratio	BG	Refs
Ti	110,000	0.3	-	Zhang et al. (2018c)
PEEK	3,500	0.3	-	Zhang et al. (2018c)
ZK60	44,660	0.305	**+**	Sun et al. (2021)
A/W	32,000	—	**+**	Bozkurt et al. (2018)
Porous Ti				
Porosity 65%–80%	675–2,653	0.3	**+**	Zhang et al. (2018c)
Porosity 0%–81%	3,700–118,700	0.31–0.42	**+**	Song et al. (2021a)

BG: bone growth; Ti: Titanium alloy; PEEK: polyether ether ketone; ZK60: Magnesium alloy; A/W: Apatite-Wollastonite bioceramic composite; **+**: positive; -: negative.

Because of its biological compatibility, magnesium alloy is considered a potential material for orthopedics. Currently, mechanical research on magnesium alloy cages focuses primarily on screws and plates, with little investigation on the cage itself. In an FEA study, the biomechanical properties of the cervical cage based on magnesium alloy ZK60 were evaluated, and the microporous structure of the magnesium alloy cage was enhanced utilizing lattice topology optimization technology (Sun et al., 2021). The findings demonstrated that the new cage may further reduce stiffness and stress shielding while still providing appropriate space for bone growth. These discoveries might aid in the design and manufacture of future magnesium alloy cages.

The Apatite-Wollastonite (A/W) bioceramic composite is a bioactive and compatible material used for hard tissue repair; whether it can withstand enough physiological loading to be used in ICs is of great interest. The biomechanical behavior of a novel apatite-wollastonite interbody cage was evaluated in a finite element model of the lumbar spine (Bozkurt et al., 2018). The findings provide favorable support for the A/W bioceramic composite as an effective material used for interbody fusion.

**FIGURE 3:**
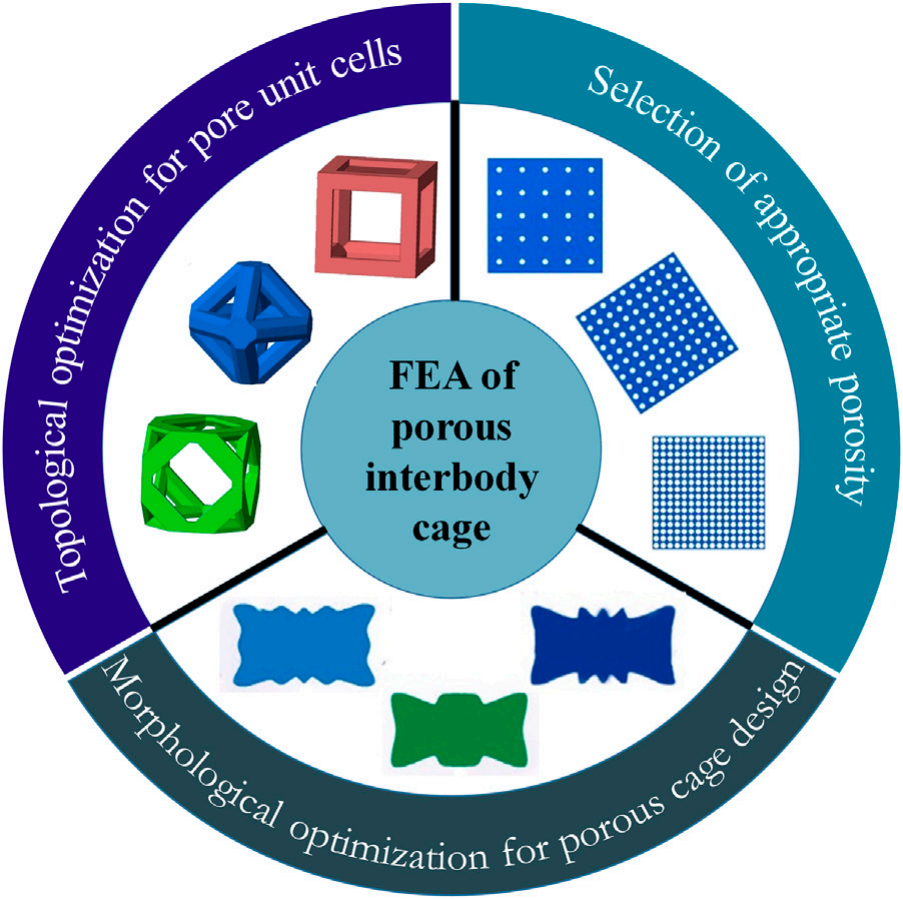
A schematic diagram of the classification of FEA of the porous interbody cage.

The porous cage is a type of special material cage that has attracted a lot of attention due to the advancement of additive manufacturing (AM) technology. Porous structures, which are promising for orthopedic implants, may be customized to produce the appropriate mechanical properties by altering the volume fraction of the topology. Furthermore, the interconnected porous architecture promotes nutrient transport and bone tissue ingrowth (Zhang et al., 2018c; Zhang et al., 2018d). Many FEA investigations have made advances in terms of pore unit cells, porosity, and porous cage optimization (as shown in Figure 3).

The topology of the pore unit cells and porosity are the fundamentals of a porous material, which may influence its elastic and shear modulus mechanical properties. The FEA can assess the structural differences between several topologies and provide the best acceptable theoretical topology with appropriate mechanical properties for bone fusion. A numerical study used FEA to prototype a 3D printed lattice embedded in an interbody cage for spinal fusion treatments and developed a computational approach to analyze property trade-offs such as beam diameter for a given topology (Egan et al., 2017). The proposed method may be utilized to evaluate lattice properties analytically through interpolation from simulated data, which is useful in the design of porous cages.

Porosity-related research focuses on determining the optimal porosity by investigating the biomechanical effects of porosity on porous structures. A surgical finite element (FE) model was developed to assess the biomechanical differences between fully porous cages, partial porous cages, and solid cages (Zhang et al., 2018c; Zhang et al., 2018d). The results showed that a porous cage with a porosity of 65%–80% had advantages in cage stress and end plate stress and may offer an alternative to PEEK cages. Another study used FEA to assess the safety and suitability of porous cages with porosities ranging from 0% to 81% (Song et al., 2021a). According to the simulation results, the behavior of spinal tissue and implants was most stable at porosities of 40%–60%. A recent study used finite element simulations to compare the biomechanical effects of three different porosities (12.5%, 41.2%, and 80.8%) with and without bone fusion (Chen and Chang, 2021). According to the findings, the porosity of the porous cage is important for contact pressure on the bone surface and cage stress. Higher porosity cages are recommended to reduce contact pressure, while lower porosity cages are recommended to replace larger loads. It is worth noting that the porosity selection and optimal porosity results in the aforementioned studies differ, indicating that there is no systematic methodological strategy for determining the appropriate porosity in porosity studies at the moment. As a consequence, more research is needed to explore the differences of the most available porosity in various studies.

Porous cage optimization typically employs computational algorithms to optimize the design of cage morphology for cage subsidence. A finite element study used optimization algorithms to design a porous cage with optimal anti-subsidence morphology and simulated the PLIF surgery to evaluate the biomechanical properties of the optimal cage at various porosities (69%, 80%, and 85%) (Tsai et al., 2016). The results revealed that porosity of 69% and 80% resulted in better biomechanical performance, and the subsidence resistance of the optimum design was superior to conventional cage designs. In 2018, a porous cage optimized scheme combining multiscale mechanics and density-based topology optimization was proposed, and the simulation results suggested that the optimized cage had a lower risk of subsidence (Moussa et al., 2018). A more recent study designed a novel porous cage using a novel global-local topology optimization method to reduce the risk of cage subsidence and stress shielding (Wang et al., 2020). The ideal biomechanical effect of the optimized cage was validated by evaluating the biomechanical properties of the optimized cage in TLIF surgery simulation.

Overall, FEA is a useful tool for investigating new cage materials and choosing the optimal material to reduce cage subsidence. Besides, FEA is also used in traditional cage material research fields, such as the comparison of Ti and PEEK for lumbar spine biomechanics (Wang et al., 2021a); biomechanical validation of 3D printed composite cage materials (Lim et al., 2019; Provaggi et al., 2019); the effect of material compliance on reducing the risk of cage subsidence (Chatham et al., 2017); and the biomechanical properties of allograft materials (Kwon et al., 2020).

### 3.2 Cage design

One of the most commonly used applications in the FEA of spinal ICs is the biomechanical evaluation of a newly designed cage. For varied spine areas, the IC design has varied properties (Table 4). In cervical segments, anterior cervical decompression and fusion (ACDF) with a cage has been widely recognized for treating general cervical disc degeneration. Plate and cage construct (PCC) is a prominent approach utilized in ACDF treatment, but with a comparatively high incidence of adjacent segment degeneration (ASD). Adequate *in situ* mobility preservation or zero-profile devices were deemed effective for lowering the incidence of ASD, so the dynamic cage and zero-profile fixation system were chosen in majority new cervical cage designs.

**TABLE 4:** A brief description of IC design features in varying spine areas.

Spine area	IC design characteristic description
Cervical spine	Dynamic cage and zero-profile cage designs for ASD problems after ACDF.
Thoracic spine	Fewer studies lead to less significant features
Lumbar spine	Targeted cage design for different lumbar interbody fusion technologies

ASD: adjacent segment degeneration; ACDF: anterior cervical decompression and fusion.

**FIGURE 4:**
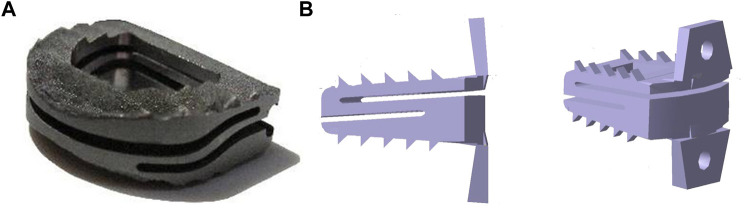
**(A)** Z-Brace dynamic fusion cage, adapted with permission from (Liu et al., 2017a). Copyright 2017, SAGE, and **(B)** S-Type dynamic zero-profile cervical cage, adapted with permission from (Manickam et al., 2021). Copyright 2021, Elsevier.

The advantages of the commercial product Z-Brace dynamic fusion cage (Baui Biotech, Taiwan. as shown in Figure 4A) in protecting the adjacent disc from over-stress and excessive mobility in the early stages after ACDF surgery were validated by FEA (Liu et al., 2017a). This cage’s ‘Zʼ shaped spring-like design allows for dynamic axial displacement and forward/backward flexion under cervical physiological loads while preventing excessive deformation under excessive physiological loads. The simulation results suggest that the “dynamic cervical cage” may provide alternative strategies in the treatment of degenerated cervical discs, but more research on clinical follow-up is needed to determine the actual influence on cervical fusion. Using an ACDF finite element model (FEM) to compare the biomechanical features of the Zero-P (DePuy Synthes Spine, Massachusetts) and the standard PCC, the average increase in range of motion (ROM) and stresses in the endplate, disc, and facet of the Zero-P were slightly lower than those of the PCC, indicating that the Zero-P provided better biomechanical responses (Li et al., 2020). Another finite element analysis on the biomechanical difference between zero-profile devices (Medtronic Sofamor Danek United States, Minnesota) and PCC yielded similar results (Hua et al., 2020). These findings may help us understand why patients using zero-profile devices have a lower incidence of adjacent segment degeneration (ASD) complications than patients using PCC. A novel S-Type dynamic zero-profile cervical cage was designed and evaluated by combining the characteristics of the dynamic cage and the zero-profile system (as shown in Figure 4B) (Manickam et al., 2021). In terms of segmental ROM changes and stress levels, this cage produced positive results. However, by ignoring the comparison of biomechanical behaviors between the new cage and the existing dynamic or zero-profile cages, this finding may be somewhat limited. Furthermore, an endplate-conformed cervical cage was designed to be compared to a typical non-conformed cage, and FEA results revealed that when compared to non-conformed cages, endplate-conformed cages significantly reduce cage-endplate interface stress and provide a more uniform stress distribution in both the fused segment and the adjacent segments, indicating their potential to reduce cage subsidence and protect adjacent segments from degeneration (Zhang et al., 2016a).

Unlike cervical fusion, which is primarily ADCF, lumbar fusion has a variety of fusion techniques with distinct characteristics, which may necessitate different cage design strategies. A novel narrow-surface PEEK cage was designed and its biomechanical response was evaluated for full endoscopic oblique lateral lumbar interbody fusion (FE-OL-LIF) (Ling et al., 2019). According to the FEA results, this endoscopic-based narrow surface design was strong enough to withstand the load of lumbar activities, which may provide a potential way to study the cage design of other full-endoscopic lumbar fusion techniques. The FEA was used to evaluate the biomechanical properties of a redesigned cage for crenel lateral interbody fusion (CLIF), which was modified from lateral lumbar interbody fusion to reduce approach-related complications (Chen et al., 2020). The simulation showed that the redesigned cage was effective as an alternative choice for patients with good bone mineral density (BMD) and that the length of the cage should cover the epiphyseal ring to reduce cage subsidence. To evaluate the mechanical effect of cross-bridging on the maximal stiffness of TLIF, a newly designed cage with four anterior holes in an ogival shape was introduced, allowing communication between the anterior extracage grafted bone and the intracage grafted bone (Lee et al., 2021). According to the FEA results, the anterior hole allows bony bridging between anterior extracage grafted bone and intracage grafted bone, which may increase biomechanical stability in the fused segment, indicating that the new cage may facilitate a more stable fusion process than a conventionally designed cage.

3D printed lumbar cages are also a major subject of research in the field of lumbar cage design. An anatomical 3D-printed lumbar cage for lumbar interbody fusion was developed using computational and experimental analysis combined with the 3D-printing technique, and preliminary cell culture results showed promising results in terms of cell growth and activity, confirming the construct’s biocompatibility (Serra et al., 2016). This study proposed a novel design optimization strategy based on computational and experimental analysis combined with 3D printing technology to develop anatomically shaped lumbar cages, which may pave the way for further research into 3D printing technology for customizable implants for various medical applications. A case report study comparing patient-specific 3D printed *versus* off-the-shelf implants for L5 *En-Bloc* vertebrectomy found that using a 3D printed cage reduced operative time significantly and allowed a superior anatomical fit for the patient, with the potential to improve anatomy restoration (Mobbs et al., 2018).

There have been few FEA studies of thoracic ICs. Yu et al. (2020) compared the biomechanical characteristics of two transforaminal thoracic interbody fusion cages based on the thoracic anatomy of 150 patients in the Chinese population, and their findings revealed that the kidney-shaped cage is more stable and experiences less stress after thoracic intervertebral fusion, and it is more suitable for Chinese transforaminal thoracic interbody fusion.

The application of FEA in cage design encompasses spinal fusion situations from the cervical to the lumbar. Meanwhile, FEA technology can evaluate cage design from a variety of perspectives, such as comparing old and new designs, examining effects under different fusion techniques, and creating patient-customized 3D-printed cages, demonstrating its broad application scenarios and high application potential.

### 3.3 Fixation devices

Stand-alone (SA) cage fusion has been linked to a low risk of post-treatment trauma or bleeding, as well as good stability and rapid recovery. However, complications associated with this technique, such as endplate fracture and cage subsidence, have been frequently reported (Fang et al., 2020). As a result, fixation devices were developed to avoid the drawbacks of SA cages. The pedicle screw is the most commonly used fixation instrument, and lumbar interbody fusion with pedicle screw fixation (PSF) has emerged as the gold standard treatment for lumbar spine disease. The FEA was used to investigate the effects of the PSF system on the biomechanical properties of the fusion lumbar spine, and the results confirmed that the PSF system may reduce the risk of subsidence and cage failure under both static (Zhang et al., 2018b; Fang et al., 2020) and whole-body vibration (WBV) (Wang and Guo, 2021) conditions.

The PSF system is typically made up of trajectory screws and rods. The traditional trajectory (TT) screw, which engages more cancellous bone than cortical bone, may be susceptible to screw loosening, especially in patients with osteoporosis, resulting in loss of correction and non-union. The cortical bone trajectory (CBT) screw is a new technique that may offer an alternative option. According to some recent FEA studies, the CBT has slightly better stability, less endplate strain, and less facet joint stress at the fusion segment than the TT (Su et al., 2021; Zhang et al., 2021). Furthermore, because of its diverging trajectory, CBT may have advantages in reducing the risk and incidence of postoperative radiculitis and nerve root injury, as well as avoiding more soft tissue exposure and further damage to paravertebral muscles, facet joints, and joint capsules, which promotes lumbosacral interbody fusion (Han and Wang, 2020).

The traditional rod in the PSF system is made of Ti, which can cause abnormal changes in load transfer and stress, resulting in intervertebral disc and bony structure degeneration. Furthermore, high rigidity may cause greater deformation and higher stresses in adjacent segments, leading to adjacent segment diseases (ASDs). As a result, flexible rods made of compliant materials such as nitinol, polyetheretherketone (PEEK), or biodegradable materials were introduced into the PSF system in the hope of reducing the stress-shielding effect and preventing the ASDs. According to the FEA simulations, the flexible rod system reduced stress responses in the pedicle screws but increased stress responses in the cage, implying that the flexible rod system may reduce the risk of pedicle screw breakage but increase the risk of cage subsidence and rod breakage (Fan et al., 2019). In addition, flexible rods may reduce the relative increase in contact force at adjacent facet joints and provide less stress shielding, which may slow the progression of ASDs (Tsuang et al., 2017; Hsieh et al., 2020).

Lumbar interspinous spacers (IPSs) have gained popularity as an alternative treatment for lumbar degenerative disease because they may avoid the drawbacks of the PSF technique at adjacent segments. Several recent FEA studies compared IPS implantation to PSF implantation, and the results revealed that IPS had a similar stabilization effect at the surgical segment and fewer biomechanical changes at the adjacent segments than PSF, which may be useful in preventing ASDs (Bae et al., 2020; Fan et al., 2021a). Furthermore, the effect of the IPS on the dynamic characteristics of the lumbar spine was evaluated, and the simulation result revealed that the IPS can absorb vibration energy while maintaining spine stabilization (Yin and Guo, 2021). Given that IPS is still a less invasive technique, it could be a promising candidate for spine fusion stabilization devices.

Rather than studying ASDs by improving fixation techniques, some studies have focused on simulations of the effects of removing screws and rods after fusion on the development of ASDs. According to the simulation results, removing the spinal fixator after complete fusion reduced intradiscal pressure and facet contact forces at adjacent segments and thus could be recommended as a viable option for effectively mitigating ASD progression (Hsieh et al., 2017; Tsuang et al., 2019; Hsieh et al., 2020). When ASDs occur, posterior extension revision surgery is the most commonly used treatment strategy. However, this technique necessitates reopening the previous scar and may result in more complications than primary surgery. Thus, the lateral lumbar interbody fusion (LLIF) strategy with lateral screws was proposed for ASD reoperation, and its biomechanical responses were compared to the traditional posterior lumbar interbody fusion (PLIF) strategy with a posterior extension of bilateral pedicle screws (Liang et al., 2020). The findings show that posterior extension provides reliable mechanical stability and excellent protection for the interbody cage, screw-bone interface, and cage-endplate interface, but LLIF supplemented with lateral screws may be an alternative reoperation surgical option for the treatment of ASDs.

The FEA has also been applied to other fixation problems, including the stability evaluation of oblique lumbar interbody fusion (OLIF) constructs with various fixation options (Eguchi et al., 2020; Guo et al., 2020; Song et al., 2021b), the effect of the PSF screw numbers on the spine and implant behaviors (Xu et al., 2019), biomechanical sensitivity of the cage lordotic angle (Zhang et al., 2018a) and placement (Calvo-Echenique et al., 2018), and the fixation strategy for multi-level fusion (Liu et al., 2017b; Hua et al., 2020; Lin et al., 2021).

The fixation device is typically used as an adjunct to the interbody cage in spinal fusion. While new fixation devices overcome the difficulties of traditional PSF, they also generate new flaws (as shown in Table 5). Therefore, there is no ideal fixation device yet. With new fixation devices being continuously produced and tested, FEA, as an effective method for bio-mechanical assessment, will continue to provide enough theoretical backing for their clinical application.

**TABLE 5:** Brief summary of advantages and disadvantages among different fixation devices.

Fixation devices	Advantages	Disadvantages	Refs
PSF	-Reduce the risk of subsidence and cage failure compared to SA cage	-Screw loosening, especially in patients with osteoporosis	Zhang et al. (2018b); Fang et al. (2020); Wang and Guo (2021)
		-High rigidity, may cause intervertebral disc and bony structure degeneration and ASD	
PSF(CBT)	-Better stability than traditional PSF	-Increase the progression of ASD	Han and Wang (2020); Su et al. (2021); Zhang et al. (2021)
	-Reduce the risk and incidence of postoperative radiculitis and nerve root injury		
PSF(flexible rod)	-Reduce the risk of screw breakage	-Increase the risk of cage subsidence and rod breakage	Tsuang et al. (2017); Fan et al. (2019); Hsieh et al. (2020)
	-Slow the progression of ASD		
IPS	-Slow the progression of ASD	-Less stabilization at the fusion segment	Bae et al. (2020); Fan et al. (2021a); Yin and Guo (2021)
	-Better dynamic characteristic		
	-Less invasive technique		

PSF: traditional pedicle screw fixation made up of trajectory screws and rods; CBT: cortical bone trajectory screw; IPS: lumbar interspinous spacers.

### 3.4 Bone formation/remodeling

Bone formation is critical for achieving a solid interbody union following spinal fusion surgery, and bone formation prediction may play an important role in making preoperative plans and developing spinal surgical devices. Since Wolff introduced Wolff’s law, various computer models of the bone adaptation process have been developed, the most widely accepted of which is the mechanostat principle, which links mechanical strains to bone formation and resorption. These models have been utilized in many spine-related investigations. Badilatti et al. (2015) reviewed the computational modeling of bone augmentation in the spine for vertebroplasty and concluded that bone remodeling models have great potential to give better insight into the augmentation procedure, the stability after augmentation, and the long-term consequences on bone biology. Ganbat et al. (2016) investigated the effect of mechanical loading on heterotopic ossification (HO) in cervical total disc replacement (TDR) and found that HO formation might have a role in compensating for the non-uniform strain energy distribution, which is one of the mechanical parameters related to the bone remodeling after cervical TDR. Rijsbergen et al. (2018) effectively created a coupled and patient-specific mechanoregulated model to predict disc generation and changes in bone density after spinal fusion and allow us to understand the influence of surgical intervention on the adjacent tissue remodeling. Calvo-Echenique et al. (2019) assessed the pro- and anti-osteogenic mechanical consequences of nucleotomies for the intervertebral space, and the simulation results indicated that fusion may be self-induced by controlling the mechanical stabilization without the need for additional fixation. All of the preceding research illustrates the broad utilization of bone remodeling in the spine, and the related modeling approaches are also appropriate to explore the bone remodeling after the cage is implanted.

**FIGURE 5:**
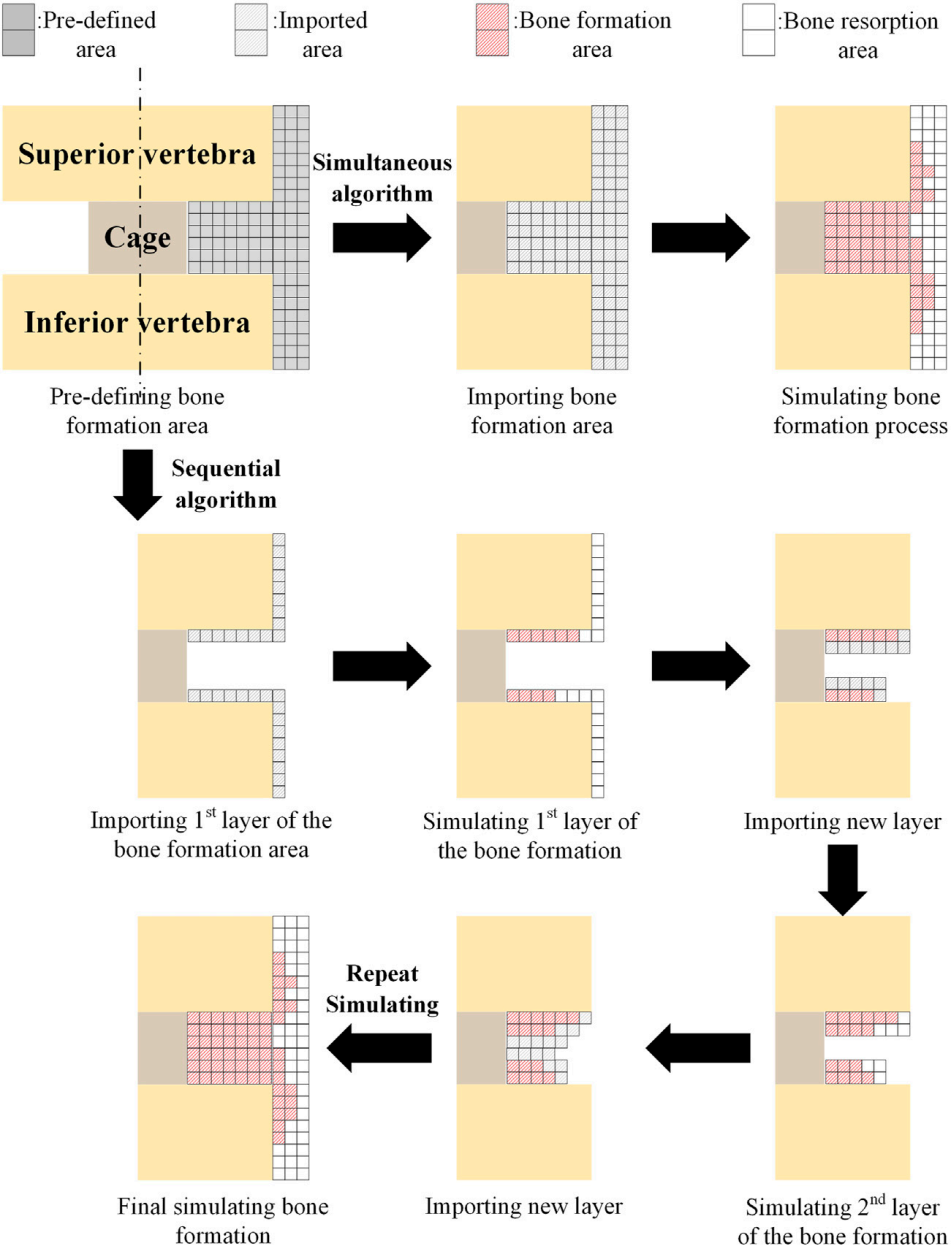
Comparison of simultaneous and sequential algorithms for simulating the bone formation. Adapted with permission from (Jin and Park, 2021). Copyright 2021, MDPI.

In simulating bone formation and resorption, two commonly used algorithm strategies are simultaneous and sequential algorithms (as shown in Figure 5). The simultaneous algorithm computes bone formation and resorption at the same time. Following the calculation, the elements of the formed bone are preserved, while the elements of the resorbed bone are removed. The sequential algorithm simulates intramembranous ossification in which layer-by-layer bone formation occurs. The elements adjacent to the superior and inferior vertebrae were imported into the predefined bone region, and bone formation and resorption were calculated as the first step of simulation. Then, the elements for the resorbed bone were erased, and the elements adjacent to the remaining elements, which were the elements of the formed bone in the previous calculation, were imported to form the new layer. After that, the bone formation and resorption were calculated again as the second step of simulation. This procedure was repeated until no new bone formation occurred (Jin and Park, 2021). The simultaneous algorithm is easier to compute, whereas the sequential algorithm is more consistent with the actual physiological situation. In both the simultaneous and sequential algorithms, the bone formation and resorption were calculated based on the mechanical stimulation of the strain energy density (SED, U ) predicted in the FEA. The density of the bone, ρ , was calculated using an equation as follows (Park and Jin, 2019; Jin and Park, 2021):

$$\frac{d\rho }{dt}=\left\{\begin{array}{c} B\left[S-\left(1+s\right)K\right]\\ 0\\ B\left[S-\left(1-s\right)K\right] \end{array}\right.\;\begin{array}{l} if\\ if\\ if \end{array}\;\begin{array}{c} S>\left(1+s\right)K\\ \left(1-s\right)K\leq S\leq \left(1+s\right)K\\ S<\left(1-s\right)K \end{array}$$

where dt , B , K , and s are the units of time, remodeling rate coefficient, homeostatic equilibrium SED, and threshold range of the lazy zone, respectively. The SED per unit mass, S , is calculated as S=U/ρ .

In practical applications, a comparative investigation was proposed using the simultaneous algorithm to quantify the differences in simulating the fusion process between the simplified axisymmetric two-dimensional (2D) model and the non-axisymmetric three-dimensional (3D) model of the lumbar segment, and the results demonstrated the critical role of the realistic 3D geometry model in predicting bone formation after lumbar spinal fusion (Hsu et al., 2018). Although extragraft bone formation (ExGBF) has been reported to be important in clinical practice, few studies have investigated ExGBF’s biomechanical effects on the motion segment. As a result, a numerical simulation of ExGBF using a sequential algorithm was proposed (Park and Jin, 2019). ExGBF has been shown to improve motion segment stability and reduce the risk of trabecular fracture in the cervical spine, demonstrating its significance. In addition, mechanical stimulus is proven to be a major factor influencing ExGBF, which in turn affects spine mechanics (Jin and Park, 2021).

In contrast to theoretical modeling studies that use complex bone formation algorithms, some studies only use bone formation as an influencing factor to investigate long-term spinal fusion stability, so bone formation modeling is typically simplified in a variety of ways. Porous cages have the advantage of allowing bone tissue to grow into the cage’s connected holes, improving long-term stability. As a result, the immediate and long-term biomechanical responses of porous titanium cages with 50% porosity in PLIF were assessed in an FEA study (Lee et al., 2016). The bone formation process was simplified by filling the pores of the cage with bone tissue, and the results confirmed the ability of cages with 50% porosity to improve the long-term stability of lumbar segments with degenerative disk disease. A material-based simulation method was proposed to evaluate the temporal bone graft changes in extreme lateral interbody fusion (XLIF) (Ramakrishna et al., 2021). This study divides the change in bone density during bone formation into four stages, with the material properties of silicone, poly (methyl methacrylate) (PMMA), cancellous bone, and cortical bone being used to simulate, respectively. The findings revealed that the load was shifted from the cage to the graft during the early stages of bone formation and then re-distributed from the ligaments and facets to the implant after complete fusion, implying that once complete fusion is achieved, the existing load paths appear to be diminished. Other simplified methods were used to investigate the long-term effects of placing one or two cages in instrumented posterior lumbar interbody fusion (Zhang et al., 2016b) and the anterior bridging bone in a newly designed cage for lumbar interbody fusion (Lee et al., 2021).

The process of bone formation is difficult to quantitatively study experimentally or clinically, thus the theoretical model of bone formation based on FEA is significant and can help to understand the long-term stability mechanism of the interbody cage after implantation. In the future, it is expected that the theoretical model will be able to simulate the growth process mechanism more accurately, whereas the simplified model, which is beneficial to the actual simulation needs of cages, will help to improve the long-term stability validation of cage material, cage design, and even the fixation devices.

### 3.5 Lumbar interbody fusion techniques

Lumbar interbody fusion (LIF) is a well-established treatment for a variety of spinal disorders. There are several surgical techniques available, including posterior lumbar interbody fusion (PLIF), transforaminal lumbar interbody fusion (TLIF), oblique lumbar interbody fusion (OLIF), anterior lumbar interbody fusion (ALIF), and lateral lumbar interbody fusion (LLIF). In terms of fusion or clinical outcomes, there is no distinct and definitive evidence that one approach is superior to another. However, using FEA, the differences in mechanical properties of different fusion techniques can be quantified to some extent, which may aid surgeons in decision-making and selecting appropriate surgical techniques for patients.

PLIF was one of the initial approaches to lumbar interbody fusion, first tried in 1940. In 1982, the TLIF was developed to reduce the risks and limitations associated with the PLIF procedure while maintaining spine stability (Fan et al., 2021b). The following fusion techniques were frequently compared to these two techniques. According to a biomechanical comparison of the PLIF, TLIF, Extreme lumbar interbody fusion (XLIF), and OLIF in a single segment lumbar fusion model, the PLIF had less stability than the other three fusion procedures due to greater ROM and stress peaks in the posterior instrument (Lu and Lu, 2019). Furthermore, the OLIF and XLIF procedures resulted in fewer stress peaks in the cortical endplate and cancellous bone than the TLIF procedure, which was beneficial for subsidence resistance, disc height, and segmental angle maintenance. Another study used a lumbar-spine finite element model to compare the biomechanical differences between PLIF, TLIF, ALIF, and circumferential lumbar interbody fusion (CLIF/360) (Umale et al., 2021). The model revealed that ALIF is the most flexible and CLIF/360 is the stiffest, which corresponded to the findings of the *in vitro* experimental study. A numerical study compared two different minimally invasive techniques, LLIF and TLIF (Areias et al., 2020). However, FEA results revealed that neither is significantly better than the other in terms of spinal stability.

Whole-body vibration (WBV) exposure is becoming more common in our daily lives and at work, and it is almost always present in surgical spine patients. As a result, FEA studies of LIF techniques have focused on comparing different LIFs’ biomechanical responses under WBV conditions. A finite element study compared the effects of ALIF, PLIF, and TLIF approaches on the biomechanical responses of the lumbar spine to WBV, and the results showed that the endplate stresses in the TLIF model were lower than in the ALIF and PLIF models, but the TLIF generated greater stresses in the pedicle screws and longitudinal rods at the fused segment (Fan and Guo, 2019). A more recent study compared the TLIF and PLIF techniques and discovered that the stresses of the bilateral pedicle screw fixation system were higher in TLIF than in PLIF but that the endplate stresses were also higher in TLIF (Fan et al., 2021b).

The comparative study of fusion techniques using finite element analysis is also used to validate improved techniques. Percutaneous endoscopic transforaminal lumbar interbody fusion (PE-TLIF) is a TLIF modification that allows direct access to the disc with gradual tissue dilation *via* the endoscopic transforaminal posterolateral approach, resulting in less blood loss, faster recovery times, and shorter hospital stays. The simulation in the lumbar spine finite element model revealed that PE-TLIF outperforms TLIF in terms of biomechanical performance, and the surgeon is advised to implant the cage in the anterior district of the L5 vertebra’s upper endplate in the traverse direction using the PE-TLIF technique (He et al., 2021).

The FEA is extremely useful in the study of LIF techniques. The differences between LIF techniques can be seen more intuitively in the same lumbar spine model, especially in the WBV situation, which may help the surgeon better understand the technical characteristics and assist in the optimization of the surgical plan.

### 3.6 Pathological structure influence

The pathological structure may influence fusion implantation and bone fusion progression and is therefore one of the concerns in FEA of interbody cage. The most common challenge for lumbar interbody fusion might be osteoporosis, which is associated with degenerative disease, pedicle screw failure, subsidence, and vertebral fracture in the elderly population. For simulating osteoporosis, the elastic modulus of cancellous bone is set to reduce by 66%, and the elastic modulus of cortical bone, endplate, and posterior elements is set to reduce by 33%, while the elastic modulus of other structures remains unchanged. With this configuration, finite element analysis discovered that as the degree of osteoporosis increased, the maximum stress on the upper and lower en-plates of the fusion segment increased significantly, increasing the potential risk of implant subsidence in OLIF (Wang et al., 2021b). Furthermore, the surgeon must consider the limitations of the stand-alone (SA) and lateral plate-screw (LPS) fixations in OLIF, which may not provide adequate stability for osteoporotic patients (Bereczki et al., 2021; Wang et al., 2021b). Under whole-body vibration (WBV), the osteoporosis of the fused vertebrae may make the adjacent segments more unstable, increasing the risk of adjacent segment disease, subsidence, cage failure, rod failure, and lumbar instability (Wang and Guo, 2020). These findings may contribute to a better understanding of the effect of osteoporosis on the static and vibration characteristics of lumbar spine fusion, as well as provide clinical treatment references for lumbar interbody fusion and lumbar vertebrae osteoporosis.

ASD is a common pathological structure in the bone fusion process and is addressed in many fixation device studies, but mostly from the perspective of mechanical effects on adjacent vertebrae, with little direct focus on mechanical changes after degeneration. Jiang and Li (2019)investigated the changes in intervertebral motion and intervertebral disc pressure with the progression of proximal degeneration of the lumbar spine after fusion surgery by reducing the height to simulate different degrees of degeneration. The results demonstrated that as intervertebral disc degeneration in adjacent segments progresses, the ROM of adjacent segments decreases while the nucleus pulposus and annulus fibrosus pressures increase.

In comparison to animal models and clinical studies, building pathological structures using FEA model is unquestionably a more practical and effective choice. However, given the complexity and asymmetry of pathological structures, comprehensive reconstruction is challenging owing to meshing accuracy and computational efficiency limits, resulting in many pathological structures that cannot be simulated or must be simplified. It is hoped that as FEA technology progresses, the aforementioned concerns will be addressed.

## 4 Discussion

Despite advances in finite element modeling for the study of spinal ICs, there are still numerous limitations that are frequently mentioned in discussions. A total of 50 papers were counted, and the seven items with the highest frequency of discussion are shown in Table 6. First, most FEA studies used only one unique spine model, so the simulation results may not be representative of the average person. However, as a standard technique for current finite element studies of spinal ICs, it is widely assumed that the predicted results would not differ significantly, but more experimental validation is required to support the application to complex clinical situations. Second, because many finite element models did not include real pathological spine characteristics like degeneration, disc dehydration, decreased disc height, ligament hypertrophy, osteoporosis, and spinous process fracture, there may be a difference between the actual clinical trial and the FE models. One reason is that some models were created using medical images of a healthy subject with no spine pathology, and the other is that these pathological structures must be simplified due to software limitations and computational efficiency. Third, it is difficult to solve non-linear calculations in finite element analysis, so in order to improve calculation efficiency, the majority of studies made the necessary assumption that the material properties of the spine were linear elastic, although the material properties of spinal components are non-linear in reality. The comparative study proved that the trend of deformation may be similar between linear and non-linear material models, but non-linear materials would lead the spine to be more flexible than linear ones in the simulation, indicating more realistic load conditions can be evaluated (Lan et al., 2013). Therefore, the non-linear model is superior to the linear model for FEA of the cage when the computing power is adequate. Fourth, the paravertebral soft tissue cannot be precisely recreated, and muscle function is ignored, a problem shared by numerous finite element studies. Although the lack of musculature in the models was mitigated to some extent by using the follower load technique, the complex contributions of muscles to spinal responses could not be completely replaced by the compressive follower preload. Fifth, because the nodes and elements for meshing the segment and cage had exhausted the vast computing capacity, only single-level interbody fusion, which is quite common in the FEA of the spine, was simulated. Sixth, the loading conditions were not truly physiological because, on the one hand, the models could not simulate the mechanical effect of muscle contraction and, on the other hand, the models could not evaluate the combined moments of multiple physiological loading conditions of flexion, extension, torsion, and lateral bending. Finally, intervertebral fusion can result in adjacent segment degeneration (ASD). However, biomechanical changes in adjacent segments were not taken into account in many FEA studies.

**TABLE 6:** Summary of limitations in the finite element studies of spinal ICs.

No.	Limitations	Description
1	Unique spine model	Spine modeling data is usually from individual patients or healthy volunteers, and the inter-individual variation of bone geometry and material properties does not accurately reflect the behavior of all the human specimens tested
2	Spinal pathology structure absence	Most FE models did not include real-life spine characteristics such as degeneration, disc dehydration, reduced disc height, ligament hypertrophy, osteoporosis, and spinous process fracture, so there may be a difference from the actual clinical trial
3	Linear elastic material assumption	The majority of studies that used FEA on the spine assumed that the material properties of the spine were linear elastic in order to improve computational efficiency, although the components of the lumbar spine are non-linear in reality
4	Tissues and muscles neglect	The complex soft tissue environment around the spine, including paraspinal muscles, was neglected
5	Single-level fusion	Only single-level interbody fusion was considered in the simulation because excessive calculations for the number of elements and nodes would have been required if the other spinal segments had been considered
6	Loading condition	The loading conditions were not representative of truly physiological loading conditions
7	Adjacent segment degeneration	Intervertebral fusion may cause adjacent segment degeneration (ASD). However, biomechanical changes in the adjacent segments were not considered in many FEA studies

**FIGURE 6:**
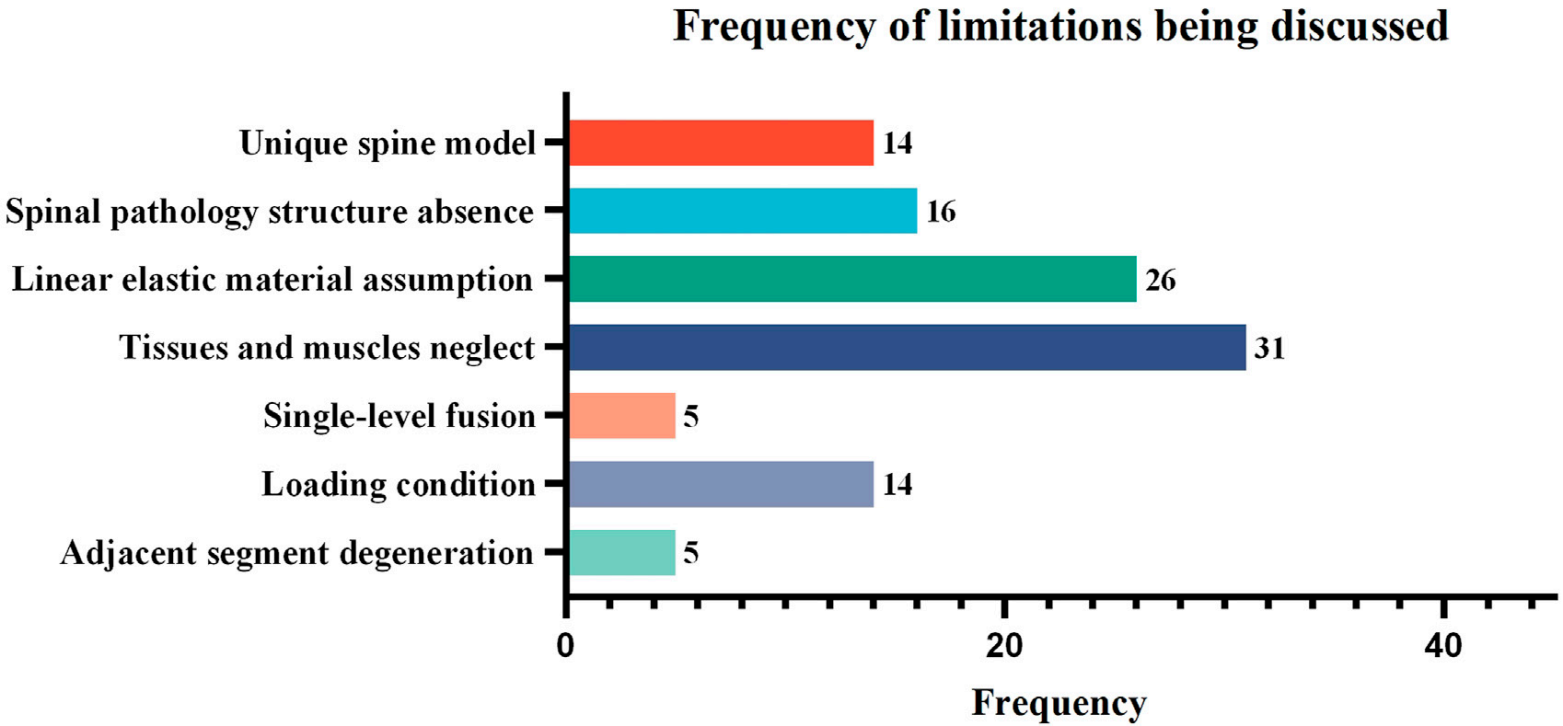
The frequency of limitations being discussed in the finite element studies of spinal ICs. Studies that did not provide a detailed discussion of limitations or that discussed highly specific rather than common content were excluded, leaving 50 articles for statistical analysis.

Among the discussed limitations, “Linear elastic material assumption” and “Tissues and muscles neglect” are the most discussed, with counts of 26 and 31, respectively. The “unique spine model,” “spinal pathology structure absence,” and “loading condition” were relatively less discussed, with counts of 14, 16, and 14 respectively. The “single-level fusion” and “adjacent segment degeneration” were the least discussed, with counts of both 5 (as shown in Figure 6).

In addition to the statistical limitations, the current scenario’s limitations are also reflected in experimental validation, long-term biomechanical evaluation, and the high cost of FEA. Validation of any finite element model is vital but highly challenging. Comparison with experimental data demands careful interpretation and analysis, since validation with the basic conditions commonly utilized in most research does not always guarantee that the model will perform well with all the complicated regimes observed *in vivo*. The long-term biomechanical evaluation of the cage is particularly significant in practical clinical applications. Nevertheless, with the exception of a few studies concentrating on bone remodeling or dynamic loading features, the vast majority of the studies only evaluate static properties, which drastically decreases the practical relevance of the simulation findings to patients. The high cost of FEA manifests in a high learning cost and significant computational cost, which may restrict its future applicability. The learning cost mostly comes from the study of the numerous software applications required in the complete FEA process, but few papers give detailed descriptions of running these software applications. A graphical guide for developing a finite element model would undoubtedly lower the learning cost (Wu et al., 2021) and hence more informative and clearly accessible review articles are required. The computational cost of an FEA model is largely controlled by the mesh density, non-linear settings, and computing power. The correctness of the FEA findings necessitates the use of a very fine mesh density in the analysis, while more meshes require more computation. Hence, it is vital to strike a compromise between mesh density and computing time. Depending on the research objectives, non-linear settings in the FEA model may occur in non-linear contact situations, non-linear material characteristics, or non-linear geometry (Fagan et al., 2002). Such a non-linear model is closer to the actual situation but takes significant calculation time. As a result, most research chooses a simpler linear model to increase computational efficiency. Computing power may be boosted by adopting parallel computing or updating computing devices (workstations or cloud computing), but the extra financial cost of doing so must be carefully weighed.

**FIGURE 7:**
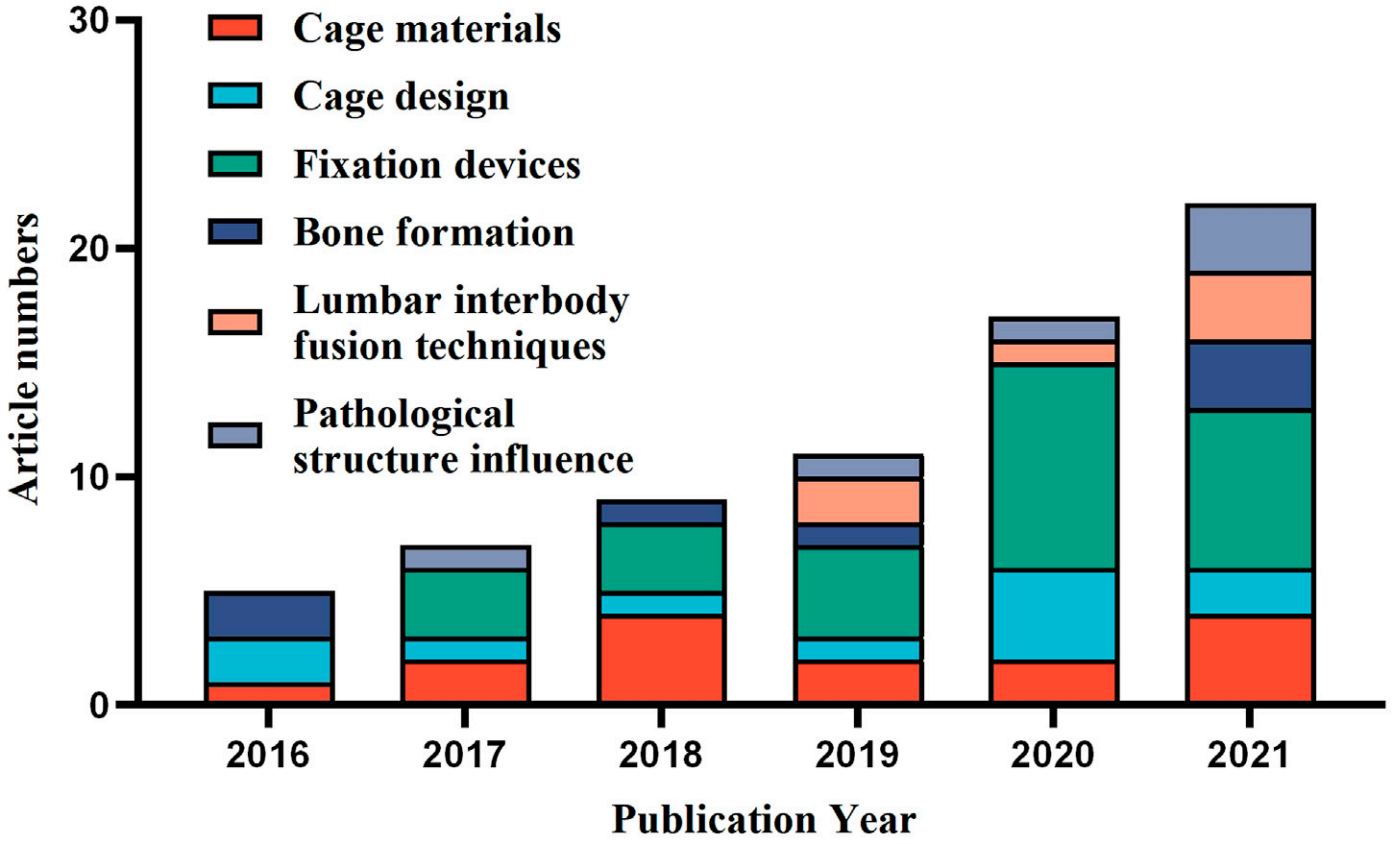
Distribution of the study classification in finite element analysis of ICs based on the publication year.

Based on the aforementioned limitations and the statistical findings of the article number of different FEA applications vs. publication year (Figure 7), the development of finite element analysis for ICs may be anticipated in the following areas: To begin with, increasing usage of public database resources is expected, which may be overcome the “Unique spine model” problem to some extent. Because such public CT image resources may considerably minimize the resource consumption of pre-modeling, more simulations can be analyzed in the same amount of time. Furthermore, the same model resources enable the comparison of the outcomes of different studies, allowing for a more objective appraisal of new findings. Second, as computational efficiency is an important factor limiting the accuracy of spinal finite element models, enhancing computing power will provide a significant boost to the FEA of ICs. Since workstation computers (Zhang et al., 2018b; Zhang et al., 2018c; Zhang et al., 2018d) and GPU-accelerated computing technology (Sun et al., 2021) have been used in some studies to improve computational efficiency, it can be expected that new computer technologies, such as cloud computing, will improve computational efficiency even more and significantly improve limitations such as “Spinal pathology structure absence”, “Linear elastic material assumption”, and “Tissues and muscles neglect”. Third, intact spine models may become the primary focus of future research. With the advancement of finite element software and computational efficiency, an increasing number of studies are being conducted with an intact lumbar spine model for simulation (Hsieh et al., 2017; Bae et al., 2020; Fan et al., 2021b; Umale et al., 2021), bringing simulation results closer to reality and supplying a foundation for studying multiple-level fusion and ASD. Ultimately, according to the results in Figure 7, with an increasing number of ICs-related FEA studies published each year over the last six years, cage materials and design will continue to receive attention in the future because of their ongoing publication. Fixation device-related FEA studies have accounted for a larger proportion of the literature in the last five years, demonstrating that its technological update pace is rapid and that it will be an important research field in the future. In the past three years, the number of studies on LIF technology and pathological structure influence has increased, indicating that it may be a potential research field for ICs-related FEA studies. Bone formation-related research is relatively rare, most likely due to the difficulty of its modeling complexity and computational efficiency, and thus new models or computational solution methods may be required in the future to accelerate its progress.

## 5 Conclusion

There have been several advancements in the FEA of spinal ICs in the last six years alone. It has provided quantitative evaluations in facilitating cage material development, optimizing cage and fixation device design, understanding bone formation mechanisms, comparing the benefits of various LIF techniques, and investigating the impact of pathological structure, which ultimately help to improve spinal fusion rates and decrease cage subsidence or migration. Furthermore, by developing public databases, future research may reduce the limitations that most applications of cage material, cage design, and fixation devices can only be verified on a unique spine model, which undeniably improves the effectiveness of model research. Concurrently, it is also hoped that with the advancement of computer technology and improved computational efficiency, more comprehensive pathological spine models will become available, contributing to the further development of bone formation, LIF technique comparisons, and pathological structural impact studies.

In such a fast-paced field, the most recent cage design, material selection, or fixation device pairing may be obsolete by the time researchers have thoroughly evaluated their efficacy. Although FEA can provide quantitative data results in the early stages, greatly shortening the evaluation process, the pathological spine component is inevitably simplified in most FEA studies due to current limitations in computational efficiency, which may differ from actual clinical trials. As a result, additional experimental and clinical validation is required.

Given the complexities and breadth of spinal surgery, one type of cage may be the best option for one spine segment or procedure but be unsuitable or sub-optimal for others. Continued research and innovation in finite element analysis of cage materials, cage and fixation device design, bone formation simulation, LIF technique comparison, and pathological structure influence assessment will help to quantitatively and visually demonstrate the cage characteristics after implanting, reducing the learning cost of surgeons for new cage products, and probably assisting them in determining the ideal ICs for patients.
